# *Ganoderma lucidum* Mycelia Mass and Bioactive Compounds Production through Grape Pomace and Cheese Whey Valorization

**DOI:** 10.3390/molecules28176331

**Published:** 2023-08-30

**Authors:** Vasiliki Kachrimanidou, Aikaterini Papadaki, Harris Papapostolou, Maria Alexandri, Zacharoula Gonou-Zagou, Nikolaos Kopsahelis

**Affiliations:** 1 Department of Food Science and Technology, Ionian University, Argostoli, 28100 Kefalonia, Greece; vkachrimanidou@gmail.com (V.K.); kpapadaki@ionio.gr (A.P.);; 2 Department of Ecology and Systematics, Faculty of Biology, National and Kapodistrian University of Athens, 15784 Athens, Greece

**Keywords:** *Ganoderma* sp., medicinal mushrooms, bioeconomy, food waste valorization, functional compounds

## Abstract

Numerous compounds obtained from the medicinal mushroom *Ganoderma lucidum* have evidenced renowned bioactive characteristics. Controlled fermentation to generate fungal mycelia confers several advantages, specifically when the valorization of agro-industrial streams as fermentation feedstocks is included. Submerged fermentation of a newly isolated Greek strain of *G. lucidum* was performed using conventional synthetic media and, also, grape pomace extract (GPE) and cheese whey permeate (CWP) under static and shaking conditions. Under shaking conditions, maximum biomass with GPE and supplementation with organic nitrogen reached 17.8 g/L. The addition of an elicitor in CWP resulted in a significant improvement in biomass production that exceeded synthetic media. Overall, agitation demonstrated a positive impact on biomass productivity and, therefore, on process optimization. Crude intracellular and extracellular polysaccharides were extracted and evaluated regarding antioxidant activity and polysaccharide and protein content. FTIR analysis confirmed the preliminary chemical characterization of the crude extracts. This study introduces the design of a bioprocessing scenario to utilize food industry by-products as onset feedstocks for fungal bioconversions to obtain potential bioactive molecules within the concept of bioeconomy.

## 1. Introduction

The controlled fermentation of medicinal fungi has been regularly exploited to generate bio-based and high-value products (e.g., enzymes, polysaccharides, proteins, and peptides) that comprise a wide range of compounds with diversified potential applications. Mushroom bioactive compounds have been widely studied with respect to their health benefits, including immunomodulatory properties, antitumor, and antioxidant activities, along with nutritional value [1,2]. Mushrooms contain primarily vitamins, minerals, and essential amino acids and also indicate a major source of carbohydrates, specifically glucans, which could exert a prebiotic effect [3]. In particular, polysaccharides and polysaccharide—protein components are of paramount significance based on their proven bioactivity [4] and can be obtained from the fruiting bodies or the mycelia biomass through mild extraction processes and novel green methods [5]. It is worth noting that roughly 650 of ~2000 known edible mushrooms have evidenced medicinal characteristics [6] that highlight their prominent potential. Likewise, the annual increase (7%) in global edible mushroom production also pertains to the wide range of end applications and health benefits [7].

In general, the genus *Ganoderma*, part of the Ganodermaceae family, and specifically *G. lucidum*, also known as reishi or lingzhi, is recognized as one of the widely evaluated mushrooms worldwide and traditionally used in Chinese medicine [8]. Polysaccharides, proteins, peptides, and fatty acids found in mushrooms and extracts elicit health benefits, including antitumor, cytotoxic, antimicrobial, and antioxidant properties [8]. Given the properties of β-glucans, specific prominence has been directed to the polysaccharides of *Ganoderma*. The latter can be employed as food components or nutrient supplements, whereas several recent notices from the Food and Drug Administration (FDA) have indicated their Generally Recognized as Safe (GRAS) status (GRAS Notices 413) [9]. In fact, several studies in the open literature have reviewed the health benefits associated with the polysaccharide or protein—polysaccharide extracts obtained from *G. lucidum.* Actually, the vast majority elaborates on the extracts obtained from the fruiting bodies and, to a lesser extent, on investigations of fungal mass extracts. The latter demonstrates the advantage of shorter fermentation periods along with controlled fermentation, employing either solid-state or submerged fermentation processes [10]. Likewise, submerged fermentation promotes higher mycelia productivity, process standardization, and lower occurrence of contamination, whereas downstream processing is more straightforward [11,12].

Numerous studies undertook the investigation of several parameters during submerged cultures of *G. lucidum* and of the subsequent effect on biomass and polysaccharide formation [3,12]. Media composition (e.g., carbon and nitrogen sources, vitamins, lipids), inoculum concentration, aeration, pH value, and agitation have been evaluated [3,12]. However, these studies predominantly utilize conventional synthetic or semisynthetic supplements that contribute to elevated costs. Thus, an additional benefit could stem from the valorization of low-cost substrates, such as agro-industrial waste and by-product streams, as the sole nutrient supplement in submerged fermentations. For instance, Diamantopoulou et al. used glucose liquid cultures enriched with olive mill wastewater and evaluated biomass and polysaccharide production of *Ganoderma resinaceum* [13,14]. Other studies, reporting on the use of food and municipal waste, focus on the biomass of fruiting bodies [15]. In fact, the deployment of such bioprocesses could proclaim enhanced sustainability and facilitate the transition to a circular economy.

Within this context, this study designates the valorization of food industry by-product streams as onset feedstocks in submerged fermentation of an indigenous, newly isolated *G. lucidum* strain. More specifically, the side streams of winemaking and cheese manufacturing—namely, grape pomace and cheese whey, respectively—were evaluated as fermentation feedstock to substitute for conventional synthetic media. The selection of the aforementioned side streams was established on the basis of developing novel and sustainable concepts, which could be integrated into existing small-scale processing facilities and promote food systems’ circularity. Cheese whey indicates a highly polluting outflow, consisting primarily of high lactose, but also protein, lipids, salts, and minerals, which could be exploited via sustainable processing routes [16,17]. Likewise, grape pomace corresponds to approximately 60% of the solid by-products of the vinification process, containing skins, seeds, and stalks [18]. Moreover, sugars, nitrogen, minerals, and lipids can be obtained from grape pomace via mild treatment methods and further valorized in microbial bioconversions [19]. Therefore, our study explored the potential of these renewable resources as feedstocks for *G. lucidum* biomass and bioactive compounds, taking into consideration crude intracellular (IPS) and extracellular polysaccharide (EPS) production. The eventual goal will be to design bioprocesses that will enhance the resilience of food systems within the concept of bioeconomy.

## 2. Results and Discussion

### 2.1. Mycelium Growth Rate on Solid State Fermentation (SSF)

The first step of the experiment was performed using potato dextrose agar (PDA) as the substrate on SSF to assess the mycelium growth rate. PDA provides all the essential nutrients to support fungal growth. Radius measurements were carried out every 24 h, and the Kr of the newly isolated Greek strain of *G. lucidum* was found to be 3.88 mm/day. Kuhar et al. reported that the radial growth of *G. lucidum* ranged between 9 and 11 mm/day using malt extract agar [20], whereas the reported radial growth on PDA ranges from 3.34 mm/day [21] to 7.5 mm/day [22].

### 2.2. Growth Performance on Conventional Carbon Sources Using Complex Media

Subsequently, different carbon sources—namely, glucose, fructose, and a mix of glucose and fructose—were implemented to assess the growth performance of the *G. lucidum* strain. Fermentation kinetics were monitored up to carbon exhaustion, and biomass along with sugar consumption was measured in five-day intervals. Table 1 presents the results using glucose, fructose, and a mix of glucose and fructose during static and shaken submerged fermentations (SmF). The maximum production of total dry weight (TDW_max_), TDW productivity (Q_X_), substrate consumption rate of the process (Q_S_), and conversion yield (Y_X/S_, g/g) are presented. Generally, it can be observed that shaking conditions resulted in increased productivity (Q_X_), considering the shorter fermentation period, except for the case of glucose. In fact, the final biomass concentration in the case of glucose was lower during shaking conditions compared with static (10.45 and 11.44 g/L, respectively), but statistical analysis showed that the difference was not significant (*p* < 0.05). The application of fructose as the sole carbon substrate did not favor fungal growth, specifically in static fermentation, as evidenced by lower conversion yields in both conditions, also demonstrating an extended lag phase during the first days of incubation. Interestingly, the productivity increased more than twofold when agitation was applied, considering the well-established effect of oxygen supply on fungal growth. On the other hand, when mixed sugars were employed, the results improved compared with fructose, yielding 6.60 and 9.05 g/L of biomass in static and shaking conditions, respectively. Particularly, biomass productivity using mixed sugars (in shaking conditions) was even higher with glucose, postulating a synergistic effect of mixed sugars on fungal metabolism and growth. Likewise, biomass productivity and conversion yield in shaking conditions were superior to static conditions when fructose and mixed sugars were assessed.

The effect of agitation on increased fungal biomass production has been previously demonstrated in several *Ganoderma* isolates [23,24,25], focusing either solely on optimizing fungal mycelia proliferation or combining it with the production of extracellular or intracellular polysaccharides. Apparently, agitation confers a crucial parameter as it directly relates to oxygen transfer and shear stress, therefore with the size of pellets and secondary metabolites [3]. Hence, in the case of cultures under static conditions, it is possible that oxygen transfer is not adequate to support fungal respiratory requirements, thus entailing lower biomass production.

Previous research in the literature has indicated that mixed carbon sources proved to be beneficial for the growth and production of ganoderic acid during submerged fermentation [11,26]. Actually, Wei et al. suggested that using an optimized ratio of glucose and sucrose improved the fungal mass of *G. lucidum*, along with polysaccharide and ganoderic acid production [26]. In a recent study, Bakratsas et al. used mixed ratios of glucose and xylose during the cultivation of *Pleurotus ostreatus* for mycoprotein production [27]. The authors reported that when the combination of glucose and xylose was assessed, sugar consumption was initiated after four days post-inoculation, compared with the fastidious growth of glucose [27]. This is contrary to our study, considering that the substrate consumption rate was enhanced in the case of mixed sugars. In general, carbon type affects gene expression and the release of enzymes related to fungal growth and hyphae formation, strongly correlated with specific strains [28,29,30]. Hence, hyphae formation might be associated with the lower biomass production observed when fructose was applied as the sole substrate. Nonetheless, our results on sugar consumption were deemed promising with our hypothesis to replace the synthetic media and commercial carbon sources with a nutrient supplement obtained after mild pretreatment of grape pomace as an agro-industrial by-product.

### 2.3. Growth Behavior on Grape Pomace Extract (GPE) as Nutrient Supplement

**Figure 1:**
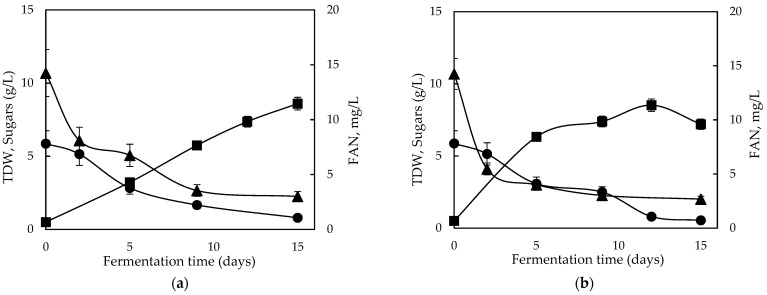
Fermentation profile of glucose (●); free amino nitrogen (FAN) consumption (▲) and total dry weight (■) production during static (**a**) and shaking (**b**) fermentation of *Ganoderma lucidum* on GPE.

Subsequent steps employed replacing commercial media with a feedstock derived from renewable resources, following the results obtained from conventional carbon sources. A preliminary characterization of GPE was performed in our previous study [31], where the low concentration of total phenolic content and free amino nitrogen (FAN) was also reported. The experiments initiated by applying GPE as the sole substrate in static and shaking conditions, and the fermentation kinetics are shown in Figure 1. Glucose consumption and biomass production started in the first five days of fermentation along with FAN consumption. Biomass productivity was higher when agitation was applied (Q_X_ = 0.68 g/L/d) compared with static conditions (Q_X_ = 0.54 g/L/d), and maximum biomass was approximately 8.6 ± 0.1 g/L in both cases, observed at different timepoints for static and shaking conditions. However, fructose was only slightly consumed in both examined conditions.

Apparently, GPE contained a significantly lower amount of organic nitrogen, considering that FAN was 15 mg/L, compared with the synthetic media; hence, in the following step, GPE was supplemented with yeast extract and peptone to simulate the synthetic substrate.

**Figure 2:**
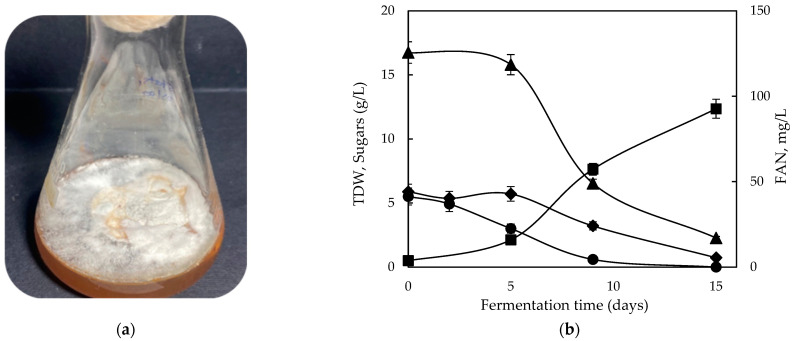
(**a**) Macroscopic observation of *G. lucidum*; (**b**) fermentation profile of glucose (●), fructose (♦), and free amino nitrogen (FAN) (▲) consumption and total dry weight (■) production during static fermentation of *Ganoderma lucidum* on GPE supplemented with yeast extract and peptone.

**Figure 3:**
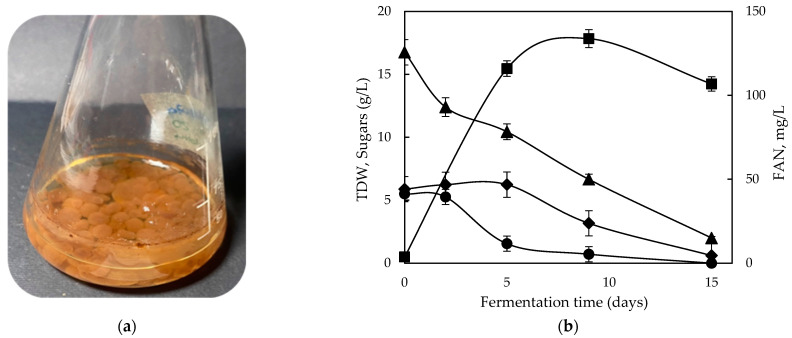
(**a**) Macroscopic observation of *Ganoderma lucidum* during shaking conditions; (**b**) fermentation profile of glucose (●), fructose (♦), and free amino nitrogen (FAN) (▲) consumption and total dry weight (■) production during shaking fermentation of *G. lucidum* on GPE supplemented with yeast extract and peptone.

Figure 2 and Figure 3 illustrate the macroscopic observation of *G. lucidum* cultures and the fermentation kinetics during static and shaking conditions, correspondingly. The maximum production of biomass was 12.4 g/L for static and 17.8 g/L for shaking conditions, obtained on the 15th and 9th day of fermentation, respectively. Notably, the supplemented GPE resulted in enhanced TDW in both conditions, compared with sole GPE and with the conventional mixed sugars case. On the other hand, in a precedent study that evaluated GPE during submerged fermentation of novel *Phellinus* sp. and *Sepedonium* sp. isolates, TDW exhibited a decrease compared with glucose; however, that study implemented only static conditions [31]. In another study, Wei et al. suggested that using an optimized ratio of glucose and sucrose improved the fungal mass of *G. lucidum*, along with polysaccharide and ganoderic acid production [26].

Agitation entailed a significant increase in process productivity in shaking conditions. The higher biomass productivity also coincided with the higher consumption rate of sugars. However, an important observation was correlated with the switch of the fungal metabolism to consume fructose, which was exhausted in both examined cases. This could emanate from the supplementation of organic nitrogen along with the oxygen transfer rates in agitated conditions that stimulated higher fungal biomass.

Overall, supplemented GPE enhanced the fermentation profile compared with conventional fermentation media, which was previously stated in the open literature. For instance, Wang et al. undertook a transcriptomic analysis of the expression of genes employed in the metabolic functions of *G. lucidum* on diversified agricultural and forestry residues (AFRs), such as rice-straw and wheat-straw, as carbon sources [29]. It is worth noting that the growth rate on glucose was lower compared with some of the AFRs, based on the results indicating that some specific genes were upregulated when agricultural by-products were used. Diamantopoulou et al. [13] reported that olive mill wastewater containing 0.8 g/L of phenolics boosted the biomass production of *Ganoderma* strains. Enhanced biomass production was also observed in the growth of *Phellinus* sp. using GPE, whereas a reduction in biomass was indicated for *Sepedonium* sp. [31]. In another study, eleven filamentous fungi, including *G. lucidum*, were grown on grape pomace under solid-state conditions to assess phenolic compound recovery and enzyme production as a biological pretreatment method [32].

In fact, *Ganoderma*, like most basidiomycetes, could benefit from biomass-derived media as these contain several complex components and resemble the natural habitat of fungi. The grape pomace used in our study contained both skins and seeds; hence, a low amount of lipids was co-extracted in GPE that might contributed to enhanced biomass production. Yang et al. also presented that different fatty acids stimulated fungal growth and polysaccharide synthesis since fatty acids could be assimilated on the cell membrane and could promote nutrient uptake [33].

### 2.4. Growth Behavior on Commercial Lactose and Cheese Whey Permeate (CWP)

Pure lactose and the lactose-rich stream obtained after cheese whey deproteinization (i.e., CWP) were also evaluated for the proliferation of *G. lucidum*. The results of the maximum total dry weight production (TDW_max_), TDW productivity (Q_X_), substrate consumption rate of the process (Q_S_), and conversion yield during static and shaking conditions are reported in Table 2. During static fermentation, the maximum biomass production was observed on the 19th and 18th day for lactose and CWP, respectively. The substitution of CWP for commercial lactose entailed a significant reduction in TDW, from 9.20 g/L to 4.90 g/L, corresponding to a decrease in the conversion yield (Y_X/S_). In order to be comparable with the lactose-based medium, CWP was diluted so that the initial concentration of lactose was ~13 g/L, resulting in a low FAN concentration, which did not sustain adequate fungal growth. Therefore, the supplementation of CWP with yeast extract and peptone was implemented, both in static and shaking conditions, similar to the GPE approach (Table 2). The addition of yeast extract and peptone had a positive effect on bioprocess productivity in both static and shaking conditions, compared with CWP. Similarly, biomass production was also enhanced compared with synthetic lactose-based media, resulting in 7.01 and 7.52 g/L for static and shaking conditions, respectively, indicating that the addition of organic nitrogen conferred a positive impact on the fermentation process.

Synthetic lactose media and supplemented CWP demonstrated higher biomass productivity in the shaking condition case, indicating a correlation with the oxygen transfer rate. Similar observations on the effect of static and shaking conditions were demonstrated by Diamantopoulou et al. for *Ganoderma applanatum* [34]. It is speculated that the lack of oxygen transfer, in combination with the fact that part of the metabolized lactose will be transglycosylated to activate the Lac operon and generate β-galactosidase, will exert an additional stress on the strain, leading to lower productivity [31]. Likewise, the study of Wang et al. demonstrated that the use of agricultural and forestry residues promoted the upregulation of laccase genes and induced fungal growth [29].

**Figure 4:**
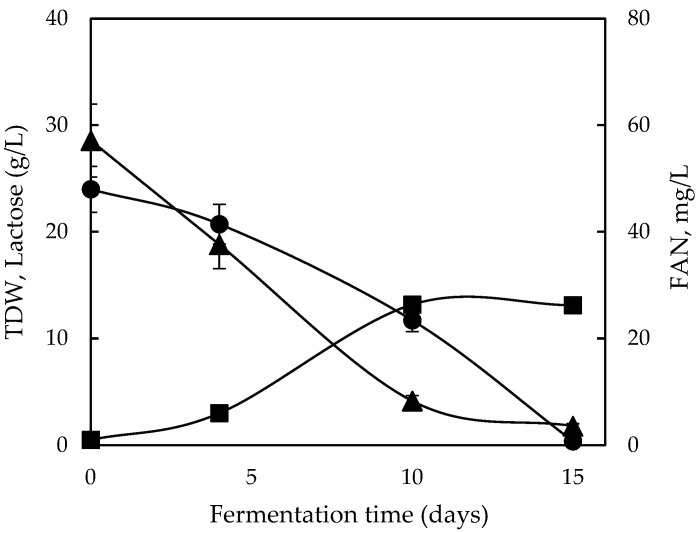
Fermentation profile of lactose (●) and free amino nitrogen (FAN) (▲) consumption and total dry weight (■) production during shaking fermentation of *Ganoderma lucidum* on cheese whey permeate with 0.25% Tween 80.

Productivity indicates a fundamental parameter under optimization as it is reciprocal to the economic feasibility of a bioprocess. Therefore, an additional experiment was undertaken, aiming to improve CWP utilization compared with the synthetic lactose-based media and enhance productivity. CWP was supplemented with 0.25% Tween 80 as an elicitor, following a previous study [35]. Fermentation was performed under agitated conditions on the basis that Tween 80 has been reported to improve oxygen transfer rate and induce metabolite synthesis [35,36]. Initial lactose was higher (~25 g/L) in an attempt to avoid both FAN dilution and supplementation with yeast extract and peptone, and the results are presented in Figure 4.

As a matter of fact, the bioprocess was substantially improved since TDW production increased almost twofold, reaching approximately 13 g/L, whereas lactose was completely exhausted until the 15th day. Evidently, the original hypothesis was confirmed as productivity (Qx) increased to 0.84 g/L/d.

Studies in the open literature that utilized either conventional lactose or cheese whey as supplements for *G. lucidum* cultivation are quite scarce to the best of our knowledge. For instance, Tang and Zhong used commercial lactose at 35 g/L and organic nitrogen sources (yeast extract and peptone) to generate 18.4 g/L dry cell weight during batch culture, which increased during fed-batch mode [37]. In the batch bioreactor experiments, lactose was exhausted after approximately 18 days, indicating a conversion yield of approximately 0.54 g_biomass_/g_substrate,_ which is in the range of the conversion yields observed in our study. An experimental design was applied to assess the use of deproteinized cheese whey (57 g/L lactose) as substrate, along with the effect of pH and temperature for *G. lucidum* growth, whereby the authors reported ~17 g/L of dry mycelia containing almost 10% of polysaccharides [38]. Whey permeate was also successfully applied during solid-state fermentation for mycelia production, using various lactose concentrations ranging between 25 and 45 g/L [39].

On the other hand, the implementation of elicitors to induce biomass or secondary metabolites formation has been previously reported, specifically for *G. lucidum* [35,40]. For instance, Zhu et al. employed the addition of fungal bio-elicitors on the 8th day of fermentation to induce biomass and ganoderic acid synthesis [40]. The authors reported that polysaccharides and proteins from *Tuber sinense* entailed an increase in cell yield when lactose was used as a substrate [40]. Yang et al. demonstrated that the addition of 0.25% Tween 80 on the 3rd day significantly enhanced fungal mass and extracellular polysaccharides production from *G. lucidum* through the stimulation of genes associated with EPS synthesis [35].

Evidently, the fermentation results of the current study are in accordance with previously published research and introduce a novel approach for the submerged fermentation of *G. lucidum* with a specific focus on the valorization of cheese whey for mycelia biomass production. To the best of our knowledge, no previous study has evaluated the application of elicitors to *G. lucidum* using cheese whey or a substrate derived from renewable resources.

### 2.5. Production and Characterization of Crude Intracellular (IPS) and Extracellular (EPS) Polysaccharides

Polysaccharides are one of the most frequently examined groups of compounds synthesized in the mycelia or the fruiting bodies of *G. lucidum* strains. These are linked with several bioactive properties as evidenced by numerous research studies in the open literature. Nonetheless, these are more focused on the biological activities of the extracts, the optimization of fungal biomass, or the utilization of agro-industrial waste and by-product streams. Bioprocess design for food waste valorization should implement a holistic approach that integrates the formulation of added-value products with potential bioactive properties.

In this context, the extraction of crude IPS and EPS was conducted in the experiments performed with GPE supplemented with yeast extract peptone and CWP with the addition of Tween 80 under agitated conditions, and the results are presented in Table 3. Extraction of crude IPS was carried out using aqueous extraction, as an established low-cost method that could be implemented in view of the potential application of the *G. lucidum* extract in food formulation.

Different patterns can be observed regarding both EPS and IPS production, depending on the carbon source. Crude IPS were higher in the case of supplemented GPE whereas the highest production occurred on the 5th day (5.94 g/L). When crude IPS were extracted from GPE (without supplementation), production was lower (2.02–3.35 g/L); still, the highest value was also achieved on the 5th day. On the other hand, when lactose from CWP was used, IPS production increased during fermentation and demonstrated the highest values (2.87–2.91 g/L) in the late fermentation stage where lactose was exhausted from the medium. Our results indicate that IPS production occurred along with carbon source utilization, as previously indicated by several studies in the open literature [34,41].

For instance, Fang and Zhong reported 1.2 g/L of IPS using conventional carbon and nitrogen sources, namely, yeast extract, peptone, and glucose [42]. Tang and Zhong assessed ganoderic acid and polysaccharide production using a fed-batch strategy, presenting a maximum production of 2.40–2.49 g/L in a shake flask and bioreactors, respectively [43]. Recently, the fermentation of a forestial *Ganoderma* isolate on a media based on potato dextrose and olive oil resulted in IPS production of 6.3 g/L [44].

Similar results were obtained for EPS production since higher values were observed within the first days of fermentation (1.28 and 3.42 g/L for GPE and CWP, respectively). In fact, EPS production from *G. lucidum* exhibits a wide range in the open literature, strongly influenced by several parameters, including carbon and nitrogen sources, pH, and agitation, among others. EPS production is also dependent on the isolation method; still, our results are in good accordance with previous studies [45]. It should be noted that crude IPS and EPS extraction was also carried out for experiments performed under static cultures, but the results were lower, advocating that agitation is beneficial for the production of these metabolites.

After the aqueous extraction, the crude IPS extracts were freeze-dried and then characterized with respect to antioxidant activity, protein, and polysaccharide content. When the antioxidant activity was assessed, the extract obtained from supplemented GPE on the 5th and 15th day caused inhibition to the DPPH• radical, yielding 12–13 μg Trolox equivalents/mg _extract_. The ABTS•+ scavenging activity confirmed the antioxidant capacity of the crude IPS extracts. A similar trend was indicated during the fermentation timepoints. A thorough investigation will be implemented in our subsequent studies to elucidate specific bioactive compounds that confer antioxidant activity.

On the other hand, crude IPS extracts from CWP also demonstrated antioxidant activity, as shown by both assays employed. Notably, the addition of Tween 80 seemed to improve the antioxidant activity of the crude IPS derived from CWP. More specifically, when only CWP was used, 3 μg Trolox equivalents/mg _extract_ were estimated, a value that increased to 5.8 μg Trolox equivalents/mg _extract_ when Tween 80 was added as an elicitor. The ABTS assay also confirmed the antioxidant activity of the extracts using CWP and Tween 80. Yang et al. also demonstrated that Tween 80 addition in the submerged cultures of *G. lucidum* entailed higher antioxidant activity [35].

The protein content in the crude IPS extract of supplemented GPE ranged between 29.3 and 37.8%, and the content of polysaccharides was 39.5–65.5%. Similarly, the protein content in the IPS extract from CWP supplemented with Tween 80 was in the range of 6–10% and polysaccharides were 32–64%, depending on fermentation timepoint.

In fact, the extracts demonstrated varying compositions regarding their polysaccharide and protein fraction, which is postulated to relate to the level of IPS purification and also to the substrate used. Our study employed a crude extract obtained after a simple aqueous treatment, and long β-glucans molecules might be branched with proteins or peptides (e.g., krestin). Hence, combined extraction methods should be preferably elaborated for a more sophisticated purification. For instance, trichloroacetic acid (TCA) could be applied to precipitate protein impurities, which could be denatured via boiling prior to extraction [46].

Likewise, the polysaccharide content is speculated to be substrate-dependent. In fact, the IPS biosynthetic pathway is regulated by specific enzymes that can be overexpressed depending on the substrate type and culture conditions [47]. The exact biosynthetic pathway has yet to be elucidated; however, numerous studies have shown that α-phosphoglucomutase (PGM), phosphoglucose isomerase (PGI), UDP-Glc pyrophosphorylase (UGD), and glucokinase (GK) are key enzymes for the regulation of polysaccharide synthetic mechanism [47,48]. Peng et al. examined the impact of mixed carbon sources on the enzymatic activities that regulate polysaccharide synthesis, showing that PGM activity was enhanced by modifying the ratio of galactose and glucose in the medium [49]. Likewise, Zhu et al. evaluated the effect of different carbon sources (among other fermentation parameters), including glucose, sucrose, and lactose on the activities of PGM, PGI, UGD, GK, and IPS synthesis during the submerged fermentation of *Cordyceps militaris* [50]. The results indicated that PGM, UGP, and PGI were significantly associated with the carbon source and reached maximum values when glucose was used, which also coincided with maximum IPS production [50].

**Figure 5:**
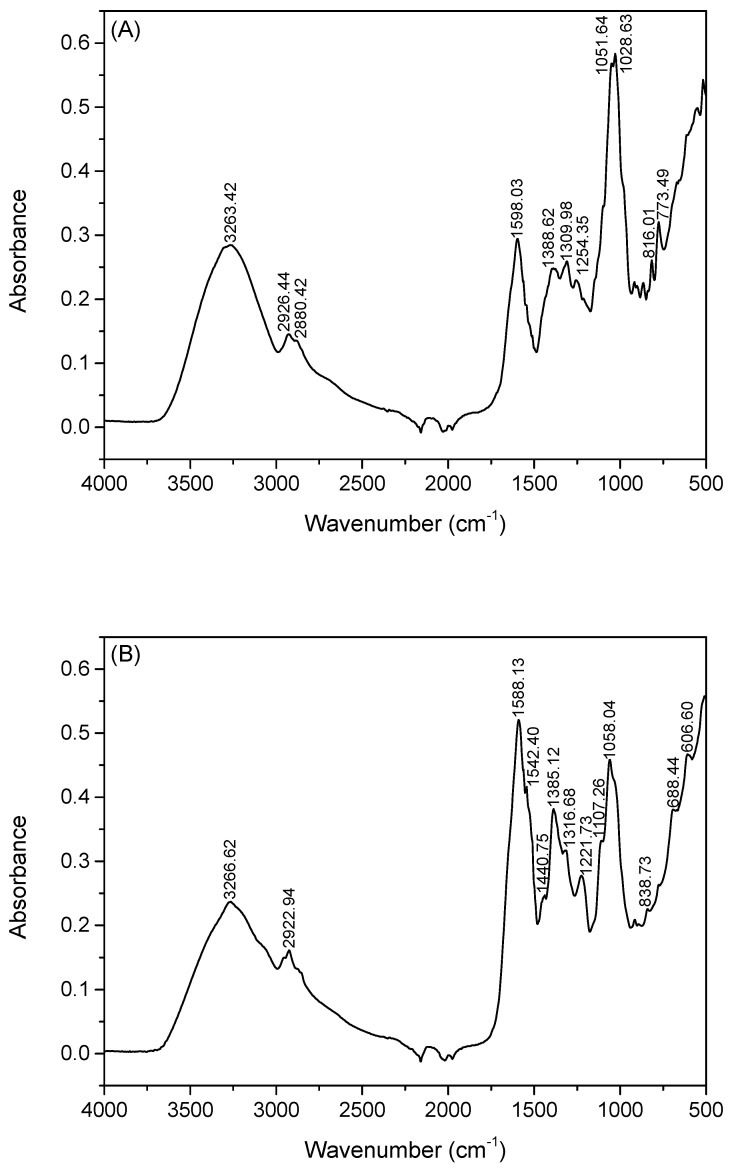
ATR-FTIR spectra of crude (**A**) EPS and (**B**) IPS extract obtained from *Ganoderma lucidum* using GPE under shaking conditions.

FTIR analysis was also performed to elucidate the spectral characterization, both for the EPS and IPS extracts obtained (Figure 5). The IR spectra from EPS (Figure 5A) demonstrated a broad-stretched peak from 3500 cm^−1^ to 3000 cm^−1^ that corresponds to the stretching vibration of the O-H group of the polysaccharide. In fact, this peak was common for IPS and EPS extracts. Nevertheless, in both cases and especially in the IPS spectrum (Figure 5B), the peak is sharper than the characteristic peak of O-H groups due to the contribution of the amide A peak (~3300 cm^−1^) of the protein that was co-extracted with the polysaccharide [51].

The bands in the region of 2500–3000 cm^−1^ can be due to the symmetric and asymmetric stretching vibrations of skeletal CH and CH2 at both EPS and IPS spectra [52]. The region of 1500–1800 cm^−1^ and specifically the band at 1598 cm^−1^ for EPS and 1588 cm^−1^ for IPS are assigned to the asymmetric stretching of carbonyl groups [53]. This peak is shifted again to lower wavenumbers, probably because of the presence of amide I and amide II of the protein. 

The bands in the region of 100–1300 are indicative of polysaccharides due to the stretching of carbonyl and C-H groups [53]. The strong peak at 1051 cm^−1^ for EPS and at 1058 cm^−1^ for IPS could be derived from the presence of C-O-C and-OH in the pyran structure. Previous studies have noted that the bands around 1041, 1153, and 891 cm^−1^ are typical for β-glucans [45]. The peak at 786 cm^−1^ could indicate α-linked glycosyl residues.

As mentioned above, some of the peaks in the IR spectra appear displaced; however, this can relate to interactions of the protein and polysaccharide complex. This could also explain the reason that amide I and amide II bands, often reported at 1626 and 1529 cm^−1^, respectively, do not appear at high shifts, regardless of the quantification with photometric methods. The IR spectra provided a confirmation for the preliminary analysis of extracellular and intracellular polysaccharide complexes.

## 3. Materials and Methods

### 3.1. Fungal Material and Culture Conditions

The fungal strain *Ganoderma lucidum*, used during this study, was isolated from a wild fruitbody of the species collected from Kefalonia island. The strain is deposited at the Culture Collection of the Ionian University, in the Laboratory of Food Chemistry and Industrial Fermentations of the Department of Food Science and Technology (CCIU 2909), as well as at the established Fungal Culture Collection of the Mycetotheca ATHUM in the National and Kapodistrian University of Athens (ATHUM 10282). The strain is maintained in PDA (Condalab) slant cultures, with or without paraffin oil at 5.0 ± 1.0 °C, at −20.0 ± 1.0 °C in 20% (*w*/*w*) glycerol, and at 5.0 ± 1.0 °C in water. Fresh mycelium for fermentation inoculum preparation was obtained after sequential growth on PDA Petri dishes, followed by a liquid preculture. Preparation of inoculum liquid precultures was performed in Erlenmeyer flasks (500 mL) filled with 150 mL of a synthetic glucose-based medium (pH 6.2) consisting of various nutrients and elements. More specifically, the liquid preculture medium consisted of glucose, 10 g/L; yeast extract, 1.5 g/L; peptone, 1.5 g/L; KH_2_PO_4_, 1 g/L; MgSO_4_·7H_2_O, 0.5 g/L; CaCl_2_·2H_2_O, 0.23 g/L; FeCl_3_·6H_2_O, 0.08 g/L, MnSO_4_·H_2_O, 0.04 g/L; ZnSO_4_·7H_2_O, 0.02 g/L (pH 6.1).

The flasks were then autoclaved for 20 min at 121 ± 1 °C and inoculated using two 10-day-old PDA agar plugs (6 mm diameter) as previously described [54]. Incubation of liquid precultures took place at 25.0 ± 0.5 °C (10 days) under agitation (150 rpm) using an orbital shaker (ZWYR-200D, LABWIT, Shanghai, China). Subsequently, the precultures were aseptically homogenized and used as inoculum (10%, *v*/*v*) for the submerged fermentation experiments.

### 3.2. Kinetic Growth Rate in Solid State Fermentation (SSF)

Solid-state fermentation (SSF) was undertaken on PDA plates (90 mm diameter) to assess the growth rate of *G. lucidum*. Briefly, a mycelium agar plug (6 mm diameter) was removed from a freshly prepared colony, aseptically inoculated at the center of a PDA plate, and placed for incubation at 25 ± 0.5 °C. A minimum of three replicates were used to investigate growth kinetics with respect to the radius mycelium growth rate. The radius growth rate (Kr, mm/day) of *G. lucidum* was estimated by measuring the colony diameter on the surface of SSF, using the equation:

$$r=\mathrm{Kr}\times t+{\;r}_{0}$$

where r and r_0_ are the colony radius at time t and t_0_, respectively, and Kr is the constant growth rate [55]. Measurements were recorded in two perpendicular directions every 24 h until the Petri dish was completely colonized by the fungus.

### 3.3. Raw Materials and Submerged Fermentations (SmF)

Submerged fermentations with *G. lucidum* were performed using conventional synthetic media with different carbon sources, specifically glucose, fructose, lactose, and a mix of glucose and fructose (i.e., mixed sugars), at an initial concentration of ~10 g/L. In the case of mixed sugars, glucose and fructose were added in equal quantities. Synthetic media contained the following (in g/L): yeast extract 2.5; peptone 3.5; CaCO_3_ 2.0; KH_2_PO_4_ 1.0; MgSO_4_·7H_2_O 0.5; CaCl_2_·2H_2_O 0.23; MnSO_4_·H_2_O 0.04; ZnSO_4_·7H_2_O 0.02; FeCl_3_·6H_2_O 0.08. Yeast extract and peptone were used as organic nitrogen sources. Subsequently, conventional media were replaced with nutrient supplements obtained from agro-industrial waste and by-product streams, namely, cheese whey and grape pomace.

Cheese whey was kindly provided by the “Galiatsatos” dairy company (Kefalonia, Greece). Prior to the fermentation process, deproteinization of cheese whey was performed to obtain cheese whey permeate (CWP), which is the lactose-rich fraction with an initial concentration of 48–50 g/L, following a previously described method [56]. The lactose-rich fraction was subsequently evaluated in the fermentation experiments after dilution to the designated concentration depending on the experiment. Grape pomace was obtained from a local vineyard, as a by-product of the vinification process of the red variety Mavrodaphni (Kefalonia, Greece), and included skins and seeds. An aqueous extraction method was applied to generate grape pomace extract (GPE) that was evaluated as a fermentation substrate. In particular, extraction of free sugars from grape pomace was carried out from the aqueous treatment of grape pomace with deionized water at 40 °C for 2 h, at a solid-to-liquid ratio of 1:10 (*w*/*v*), and contained almost equal amounts of glucose and fructose (~5–6 g/L each) [31]. GPE and CWP were independently evaluated as fermentation media, initially without the addition of any other nutrients. Specific experiments were also performed to assess the effect of exogenous organic nitrogen addition, both for cheese whey permeate and grape pomace extract, whereby yeast extract and peptone were added at 2.5 and 3.5 g/L, respectively. Last, in the case of CWP, the addition of 0.25% Tween 80 was employed as an elicitor to further improve the fermentation process.

In all the cases of submerged fermentation (SmF), Erlenmeyer flasks (100 mL) containing the designated substrate (30 mL per Erlenmeyer flask) were autoclaved (121 °C, 20 min) and inoculated with the fungal preculture (10%, *v*/*v*). In all experiments, the pH value was adjusted to 6.2 ± 0.1 using 5 N NaOH or 5 N HCl prior to autoclave.

Incubations were performed under static and agitated conditions (150 rpm) using an orbital shaker (ZWYR-200D, LABWIT, China) at 25 ± 0.5 °C. Duplicate samples were withdrawn at specific time intervals (every five days) to monitor the fermentation parameters. The experiments for all examined cases were performed twice (*n* = 2), and for each sample, the complete volume of each flask was collected (30 mL).

### 3.4. Analytical Methods

#### 3.4.1. Total Dry Weight (TDW) Determination

Samples were collected at specific time intervals (five days), and fermentation parameters were monitored. The total content of each sample was filtered under vacuum (Whatman^®^ No 1, Maidstone, UK) to separate fungal mycelia mass from the fermentation broth. The former was washed twice with deionized water, transferred in pre-weighed containers, and placed in an oven at 60 ± 0.5 °C until a constant weight was obtained. Results were expressed as total dry weight (TDW, g/L). Dried biomass was also used to study the polysaccharide content of fungal mycelia. The filtrate broth was stored at −20 °C and used for subsequent analysis of residual sugars and exopolysaccharides.

The conversion yield of the substrate to biomass (Y_X/S_, g/g) was calculated using the formula:

YX/S=(Xt−X0)(St−S0)

where X_t_ and S_t_ correspond to biomass and substrate in specific fermentation time, X_0_ and S_0_ to biomass and substrate concentrations at inoculation time.

#### 3.4.2. Sugars and Free Amino Nitrogen Quantification

Glucose, fructose, and lactose were measured by high-performance liquid chromatography analysis (HPLC, Agilent, Santa Clara, CA, USA), equipped with an ROA-organic acid H+ (300 mm × 7.8 mm, Phenomenex, Torrance, CA, USA) column coupled to a differential refractometer (RID). Operating conditions were as follows: sample volume, 10 μL; mobile phase, 10 mM H_2_SO_4_; flow rate, 0.6 mL/min; column temperature, 65 °C. Samples were diluted and filtered (Whatman^®^, Uniflo syringe filters, 0.2 μm) prior to analysis.

The concentration of free amino nitrogen (FAN) in the supernatants was measured via the ninhydrin colorimetric method as previously described [57] to evaluate nitrogen consumption.

#### 3.4.3. Extraction of Extracellular Polysaccharides (EPS)

The filtrate supernatant was treated with ice-cold ethanol at a ratio of 1:4 to precipitate EPS, following a previously described method [58]. The mixture was vigorously shaken and placed for incubation at 4 °C overnight. Subsequently, the sample was centrifuged at 4200× *g* for 10 min (Rotina 420R, Hettich Zentrifugen, Tuttlingen, Germany), and the supernatant was discarded to obtain the precipitate, which was freeze-dried to a constant weight to assess EPS gravimetrically.

#### 3.4.4. Extraction of Fungal Intracellular Polysaccharides (IPS)

Hot water extraction was performed on the mycelial biomass to obtain the crude IPS fraction of mycelia following a method previously described [59]. In particular, a specific quantity of dried mycelial biomass was extracted with boiling water at 100 °C at a solid-to-liquid ratio (1:10, *w*/*v*) for 1 h under stirring, using screw cap vials. The obtained liquid extract was filtered and freeze-dried to assess the extraction efficiency. Subsequently, the crude dried extract was resuspended in deionized water (20 mg/mL) and stored at −20 °C prior to further analysis.

#### 3.4.5. Compositional Characterization of the *G. lucidum* Aqueous Extracts

Polysaccharide content in the crude IPS extract was determined via the phenol-sulfuric method using glucose as the standard [58]. The protein content was determined using the Lowry method, and bovine serum albumin (BSA) was used for the standard curve [60]. The antioxidant activity was evaluated using both the DPPH• (2,2-diphenyl-1-picrylhydrazyl) scavenging radical method and the ABTS spectrophotometric assay. The percent inhibition (I%) of free radicals was calculated via the following equation [31]:

$$I\%=\frac{{\mathrm{ABS}}_{\mathrm{DPPH}•}-{\mathrm{ABS}}_{\mathrm{sample}}}{{\mathrm{ABS}}_{\mathrm{DPPH}•}}\times 100$$

where ABS_DPPH•_ refers to the absorbance of the blank and ABS_sample_ refers to the absorbance of the sample. A calibration curve was prepared using Trolox, and antioxidant activity was expressed as μg of Trolox equivalents per mg of dry fungal extract.

The working solution for the ABTS spectrophotometric method consisted of 2,2′-Amino-bis(3-ethylbenzothiazoline-6-sulfonic acid) diammonium salt (7 mM) and potassium persulfate (2.45 mM) and was prepared 12–16 h prior to analysis and stored in a dark cabinet, at room temperature to form ABTS•+. The solution was diluted with methanol to obtain an absorbance of 0.7 ± 0.05 at 734 nm. Subsequently, 2.85 mL of the solution was mixed with 0.15 mL of sample, and the absorbance was measured after 10 min at 734 nm. The changes in absorbance were recorded to calculate the inhibition percentage (I%).

$$\%=\frac{{\mathrm{ABS}}_{\mathrm{ABTS}•+}-{\mathrm{ABS}}_{\mathrm{sample}}}{{\mathrm{ABS}}_{\mathrm{ABTS}•+}}\times 100$$

#### 3.4.6. Fourier-Transform Infrared Spectroscopy (FTIR)

FTIR spectroscopy was performed for the structural analysis of crude EPS and IPS extracts, using an Agilent Cary 630 FTIR spectrometer equipped with diamond ATR (Attenuated Total Reflectance). The spectra were obtained after 32 scans at room temperature in the frequency range 4000–500 cm^−1^ with a resolution of 2 cm^−1^. Data were collected and processed using MicroLab PC software (Agilent Technologies, Santa Clara, CA, USA).

#### 3.4.7. Statistical Analysis

Statistical analysis was performed using Microsoft Excel 2018, and values are presented as average ± standard deviation. Student’s *t*-test was used to evaluate significant differences (significance level α = 0.05).

## 4. Conclusions

The results of this study elaborate on the growth pattern and the effect of different carbon sources and agitation conditions on the newly isolated *G. lucidum* strain. Conventional carbon sources were subsequently substituted with feedstocks derived from agro-industrial by-product streams, namely, grape pomace and cheese whey. Both raw materials sustained fungal growth, but to a lesser extent compared with commercial media; hence, the supplementation with yeast extract and peptone was assessed. Supplemented GPE entailed the highest fungal biomass production, specifically in shaking conditions, whereas biomass production was improved in the CWP case as well. On top of that, the addition of Tween 80 as an elicitor in CWP significantly improved the fermentation process. Crude IPS were extracted and analyzed for their antioxidant activity, polysaccharide, and protein content, and the estimation of crude EPS was also performed. Shaking conditions demonstrated higher productivity for EPS and IPS. FTIR analysis evidenced the polysaccharide structure of the IPS and EPS. The results of this study could contribute to the development of a bioprocessing concept that will valorize food industry by-products as nutrient sources for fungal mycelia production with potential bioactive molecules, coinciding with the concept of circular bioeconomy.

## Figures and Tables

**Table 1:** Maximum total dry weight (TDW, g/L) production, biomass productivity (Q_X_, g/L/d), sugars consumption rate (Q_S_, g/L/d), and conversion yield (Y_X/S_, g/g) during static and shaking SmF on synthetic media.

Carbon Source	Glucose	Fructose	Mixed Sugars
**Static conditions**			
TDW_max_ (g/L)	11.44 ± 0.63	5.87 ± 0.18	6.60 ± 0.27
Q_X_ (g/L/d)	0.73 ± 0.02	0.27 ± 0.06	0.30 ± 0.10
Q_S_ (g/L/d)	0.78 ± 0.04	0.44 ± 0.11	0.39 ± 0.02
Y_X/S_ (g/g)	0.94 ± 0.04	0.61 ± 0.01	0.77 ± 0.03
**Shaking conditions**			
TDW_max_ (g/L)	10.44 ± 0.52	6.18 ± 0.01	9.05 ± 0.59
Q_X_ (g/L/d)	0.67 ± 0.03	0.57 ± 0.14	0.86 ± 0.06
Q_S_ (g/L/d)	0.78 ± 0.04	0.92 ± 0.21	0.96 ± 0.23
Y_X/S_ (g/g)	0.85 ± 0.04	0.62 ± 0.01	0.82 ± 0.03

**Table 2:** Maximum total dry weight (TDW, g/L) production, average and maximum biomass productivity (Q_x_, g/L/d), and consumption rate of lactose (g/L/d) during static and shaking SmF on synthetic media and cheese whey permeate (CWP).

Carbon Source	Lactose	CWP	CWP Supplemented
**Static conditions**			
TDW_max_ (g/L)	9.20 ± 0.52	4.90 ± 0.17	7.01 ± 0.07
Q_X_ (g/L/d)	0.46 ± 0.14	0.25 ± 0.11	0.54 ± 0.08
Q_S_ (g/L/d)	0.61 ± 0.09	0.63 ± 0.26	0.86 ± 0.28
Y_X/S_ (g/g)	0.76 ± 0.04	0.40 ± 0.03	0.47 ± 0.02
**Shaking conditions**			
TDW_max_ (g/L)	8.60 ± 0.45	3.90 ± 0.44	7.52 ± 0.28
Q_X_ (g/L/d)	0.68 ± 0.12	0.20 ± 0.12	0.70 ± 0.09
Q_S_ (g/L/d)	0.87 ± 0.11	0.64 ± 0.32	1.20 ± 0.30
Y_X/S_ (g/g)	0.79 ± 0.14	0.31 ± 0.02	0.58 ± 0.09

**Table 3:** Production of crude EPS, IPS, and IPS content during SmF of *Ganoderma lucidum* on supplemented GPE and CWP with Tween 80 addition under shaking conditions.

Fermentation Time (Days)	EPS Production (g/L)	IPS Production (g/L)	IPS Content (mg/100 mg Biomass)	μg Trolox Equivalents/mg Extract *	% Inhibition **
**GPE supplemented with yeast extract and peptone**					
5	1.28 ± 0.14	5.94 ± 0.88	44.15 ± 0.27	12.72 ± 0.31	63.6 ± 0.5
15	0.50 ± 0.15	5.35 ± 0.11	39.80 ± 0.13	13.41 ± 0.52	95.2 ± 0.2
**CWP with 0.25% Tween 80 addition**					
4	3.42 ± 0.16	0.20 ± 0.02	6.97 ± 0.35	3.42 ± 0.22	59.7 ± 0.2
10	3.28 ± 0.13	1.61 ± 0.07	11.69 ± 0.41	5.86 ± 0.16	81.1 ± 0.3
15	3.02 ± 0.11	2.88 ± 0.11	22.03 ± 0.28	4.43 ± 0.19	67.8 ± 0.2

* Refers to IPS extracts measured with DPPH assay, ** refers to IPS extracts measured with ABTS assay.

## Data Availability

All data generated or analyzed during this study are included in this published article.
